# External auditory exostoses in wind-dependent water sports participants: German wind- and kitesurfers

**DOI:** 10.1007/s00405-021-06939-7

**Published:** 2021-06-19

**Authors:** Florian Wegener, Manfred Wegner, Nora M. Weiss

**Affiliations:** 1 Institute of Sport Science, Christian-Albrechts-University Kiel, Olshausenstraße 74, 24098 Kiel, Germany; 2 Department of Otorhinolaryngology, Head and Neck Surgery “Otto Körner”, Rostock University Medical Center, Doberaner Strasse 137-139, 18057 Rostock, Germany

**Keywords:** External auditory exostoses, Windsurfing, Kitesurfing, Surfer’s ear

## Abstract

**Purpose:**

Cold water and wind are known to cause exostoses of the external auditory canal. Different prevalences in different sports have been described in the literature. The aim of this study was to investigate the prevalence of external auditory exostosis (EAE) and EAE severity in coastal German wind- and kitesurfers who are exposed to cold water and strong winds. Furthermore, influencing factors such as the total exposure time and frequency of activity as well as the correlations between symptoms and the severity of EAE were investigated.

**Methods:**

In this retrospective cross-sectional study, German non-professional wind- and kitesurfers along the North and Baltic Sea coasts were recruited between September 2020 and November 2020. Each participant was interviewed about exposure time and otological symptoms and underwent bilateral video otoscopic examination to determine EAE severity.

**Results:**

A total of 241 ears from 130 subjects were analysed. The prevalence of EAE was 75.1%. In 19.9% of the participants, severe EAE was found. Exposure time and the frequency of activity had significant effects on the severity of EAE. Compared to surfers, EAE growth seems to progress faster in wind- and kitesurfers. The number of symptoms requiring medical treatment increased when two-thirds of the external auditory canal was obstructed.

**Conclusion:**

The prevalence of EAE in wind- and kitesurfers is high. Total exposure time and the frequency of activity influence EAE growth. EAE growth occurs faster in wind- and kitesurfers than in surfers. The additional influence of wind and the evaporative cooling of the EAC are thought to be responsible. The results of this study should increase awareness of the dynamics of EAE among ENT specialists and improve patient counselling.

## Introduction

External auditory exostoses (EAE) are benign, usually bilateral and symmetrical bony growths with a broad-based origin at the tympanic ring of the external auditory canal (EAC). These irreversible lesions are characterized by laminar layers of subperiosteal bone [1, 2]. Due to the high prevalence of EAE in surfers, the term “Surfer’s Ear” was introduced by Seftel in 1977 [3]. Another less common term is “Diver’s Ear” [4]. EAE have an incidence of 3–6% in the general population [1, 5] and usually remain asymptomatic. Only with progressive development can occlusion of the external auditory canal (EAC), promoted by cerumen or foreign material, lead to clinically significant complaints of external otitis, conductive hearing loss and tinnitus [1, 6–13]. The degree of obstruction that results in clinically significant symptoms or requires medical treatment is not clearly understood [7, 14, 15]. Some authors claim an obstruction of at least 80% of the EAC to be relevant [1, 5, 16, 17]. If the symptoms cannot be treated conservatively, surgical treatment may be required [1, 18], but surgery does not prevent recurrences [8, 13, 19–21]. Although the pathogenesis of EAE has not yet been conclusively explained, studies clearly indicate a relationship with frequent exposure of the EAC to water. The first water-related theories were described in 1880 by Wyman and Blake [22, 23]. Other early authors also found a relationship between EAE and water [24–26]. In 1894, Körner found that EAE occurred five times more frequently on the German Baltic coast than in central Germany [27]. In the 1930s, van Gilse and Belgraver confirmed the causative effect of cold water after finding EAE in individuals with positive “water anamnesis” [28]. Subsequently, other authors corroborated this hypothesis in swimmers [29–31] and in animal experiments [32, 33]. According to Harrison [33], the most common hypothesis regarding the aetiology of EAE is that prolonged and repeated reflex vasodilatation in the deep part of the EAC, which may be caused by cold water, causes increased vascular tension and thus triggers bone growth by increasing osteoblastic activity in susceptible individuals, possibly to protect the EAC or tympanic membrane from the cold. Further hypotheses describe the effects of chemical or mechanical irritation [1], hydration, pH imbalance, and biological factors [34] as causes of bone growth. Some authors have postulated that humans have lost the ability to protect the EAC from water ingress during evolution [1, 28, 33]. In individuals with frequent water contact, such as surfers [6–12, 14, 15, 19–21, 35–37], divers [4, 38, 39] and whitewater kayakers [16, 40], the prevalence of EAE determined by otoscopy ranges from 38.0% to 89.96% [7, 37]. In this context, the duration of water exposure correlates positively with the prevalence and severity of EAE [4, 6–9, 11, 15, 16, 20, 21, 36–38, 40]. In some studies, water temperature was also shown to affect EAE growth [4, 7, 16, 19, 21, 30, 33, 39]. The average daytime water temperature in the coastal North and Baltic Seas has varied between 4 °C in winter and 18 °C in summer over the last 10 years (Federal Maritime and Hydrographic Agency, Rostock/Hamburg, Germany). The average daytime air temperature along the coasts of the North and Baltic Seas has varied between 3 °C in winter and 19 °C in summer over the last 10 years (National Meteorological Service, Offenbach, Germany).

In addition, wind exposure and the associated increased evaporative cooling of wet skin [41] appear to also affect the appearance and growth of EAE. Fabiani et al. [42] compared individuals involved in different water sports and found the highest EAE prevalence in sailors. This result was explained by increased evaporative cooling by the wind during sailing. King et al. [43] and Hurst et al. [8] determined a significantly greater severity in the right ear in surfers. These authors suspected that there was an association with the more frequent exposure of the right ear to the prevailing wind. Other authors also believed that wind exposure affected the growth of EAE [4, 16, 19, 39, 44].

The aim of this study was to investigate (i) the prevalence of EAE, (ii) the severity of EAC obstruction and (iii) the associated symptoms in coastal German wind- and kitesurfers who are exposed to cold water and strong winds (≈ > 4 Beaufort). For this purpose, influencing factors such as the exposure time and frequency of activity as well as the correlation between symptoms and the severity of EAE were assessed and analysed.

## Methods

In this retrospective cross-sectional study, German non-professional wind- and kitesurfers were recruited at popular surfing locations on the coasts of the North and Baltic Seas between September 2020 and November 2020. The inclusion criteria were defined as follows: (1) predominant engagement in wind- and/or kitesurfing (≥ 75%) on the German coast; (2) less (≤ 25%) engagement in other outdoor water sports; (3) the clear presence of EAC; and (4) no previous bilateral surgical removal of EAE.

The study was approved by the local ethics committee of the Institute of Sport Science at the University of Kiel. Written informed consent was obtained from all participants.

Data on the total exposure time were collected in accumulated hours as calculated by the number of years and the average frequency of activity in days per week and hours per surfing session. Temporary (e.g., due to injuries) or permanent (e.g., due to work, family) fluctuations in activity were assessed during the interview and were taken into account. EAC-related symptoms (e.g., water trapping, itching, inflammation, hearing loss) in the past 12 months were collected and classified as clinically minor or clinically significant and requiring medical treatment, following Alexander et al. [6].

Both EACs were examined using a portable digital video otoscope (XION Medical GmbH, Berlin, Germany). The otoscopic images (software DiVAS Mini v3.2 by XION) were evaluated by a senior physician specializing in otology (NMW).

As in previous studies with similar diagnostic methods [8, 12, 13, 35], the severity of EAC obstruction was described using a four-point scale based on the percentage of obstruction as follows:

**Table:** 

0	no visible obstruction
1—mild	less than one-third obstructed
2—moderate	one-third to two-thirds obstructed
3—severe	more than two-thirds obstructed

### Statistical analysis

All statistical tests were selected before data collection. Statistical analyses were performed using Microsoft Excel (version 21.01, Microsoft Corporation, Redmond, Washington, USA) and SPSS (version 27, IBM SPSS Statistics, Armonk, New York, USA). The significance level was set to *p* < 0.05. If not otherwise specified, data are presented either as the means (Ms) with standard deviations (SDs) or 95% confidence intervals (95% CIs) or as the absolute numbers with percentages. To identify differences between groups, a non-parametric Pearson *X*^2^ test and Kruskal–Wallis test were used. The post hoc tests used to correct for multiple comparisons were based on the Bonferroni correction. Correlations between categorial variables were tested using Spearman rank-order correlation, and those between continuous variables were tested using Pearson’s correlation. The analysis of factors influencing the severity of EAE was performed by multivariate ordinal logistic regression after eliminating one outlier. In cases of asymmetric obstruction, the more severely affected ear was used for the analysis, as in previous studies [6–8, 16, 35].

## Results

A total of 136 subjects were assessed for eligibility. Six subjects had to be excluded based on the inclusion criteria. In total, 130 individuals (22 [16.9%] women and 108 [83.1%] men) were included in the study. The mean age was 35.5 years (SD = 10.8). A total of 43 (33.1%) participants were windsurfers only, 55 (42.3%) participants were kitesurfers only and 32 (24.6%) participants were kitesurfers with a history of windsurfing. Of all the participants, 89 (68%) were predominantly active in the Baltic Sea, 16 (12%) in the North Sea and 25 (19%) in equal proportions in the North and Baltic Seas. Table 1 shows the participants’ demographics and exposure. In total, 107 (82%) of the subjects exercised all year long, and 23 (18%) were active exclusively from spring to fall. With increasing years of exposure, the hours per surfing session increased (*r*_p_ = 0.215, *p* < 0.014). However, the days per week surfing did not increase significantly with increasing years of exposure (*r*_p_ =  − 0.100, *p* < 0.259). The EACs were analysed bilaterally in 111 subjects (85.4%). In 19 subjects (14.6%), only one EAC could be included in the evaluation. A total of 241 ears (121 left, 120 right) were analysed. Overall, EAE were found in 181 (75.1%) of the ears. Table 2 shows the frequencies of different severities. Unilateral EAE were identified in 13 out of 89 individuals (14.6%). Seventy-six individuals (85.4%) exhibited bilateral EAE. In 60 individuals (67%), the EAE exhibited symmetric severity. In 15 subjects (17%), the left ear was more affected than the right ear, and in 14 patients (16%), the right ear was more affected than the left ear. No significant difference in severity was found between the sides (*X*^2^ (3, *n* = 241) = 1.0, *p* = 0.801).

**Table 1:** Background characteristics of the 130 wind- and kitesurfers

	Range	25th–50th–75th*	Mean ± SD
Age, years	19–70	27–34–43	35.5 ± 10.8
Age at start, years	6–39	15–19–26	20.5 ± 7.3
Exposure time			
Total years	1–46	6–13–22	15.1 ± 10.7
Days per year	9–160	39–61–89	65.9 ± 32.1
Days per week	0.2–3.1	0.8–1.3–1.9	1.3 ± 0.6
Hours per day	1.0–4.3	1.8–2.1–2.5	2.2 ± 0.6
Total hours	59–9412	780–1655–2831	2193 ± 1916

*Percentile

**Table 2:** Severity of EAE

Severity	Left ear	Right ear	Both ears	%
Normal	29	31	60	24.9
Mild	40	33	73	30.3
Moderate	28	32	60	24.9
Severe	24	24	48	19.9
total	121	120	241	100.0

**Fig. 1:**
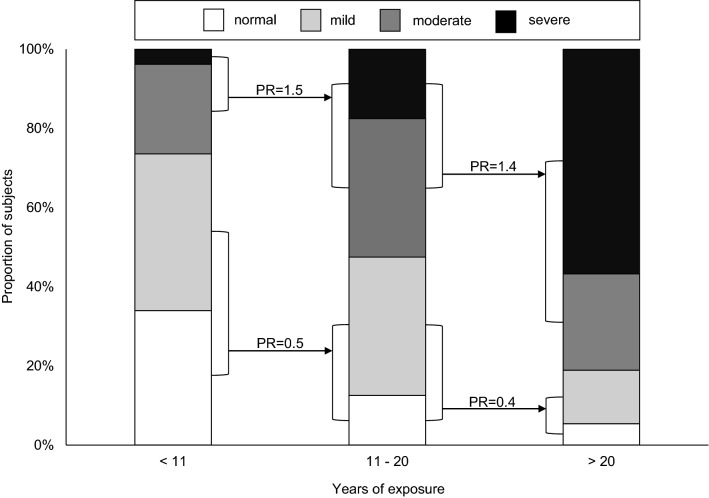
Frequencies of EAE severity according to total years of wind- and/or kitesurfing with the prevalence ratio (PR) shown paired for none + minimal and moderate + severe EAE

A significant correlation between the total exposure time and the severity of EAE was found (*r*_s_ = 0.668, *p* < 0.001). The Kruskal–Wallis test revealed significant differences in severity depending on the hours of exposure (*X*^2^ [3, *n* = 130] = 58.695, *p* < 0.001). Among 84 individuals with at least 10 years of previous windsurfing and/or kitesurfing activity, 92% (*n* = 77) had EAE. Individuals with less than 10 years of activity (*n* = 46) had a lower prevalence of EAE of 61% (*n* = 28). With each additional decade of exposure, the prevalence of moderate and severe EAE increased by 40–50%, with a simultaneous decrease of 50–60% in mild EAE and the absence of EAE (see Fig. 1).

**Fig. 2:**
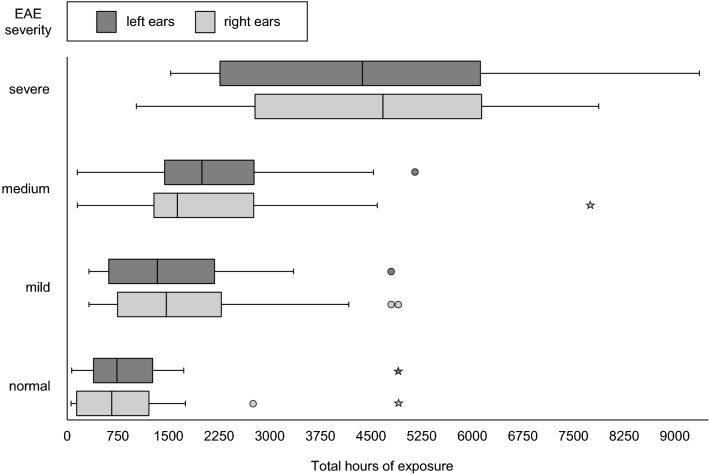
Severity of EAE according to total hours spent wind- and/or kitesurfing

The mean exposure time for individuals without EAE was 844.5 h (SD = 105.2) in 8.1 years (SD = 4.9), while it was 2514.1 h (SD = 507.3) in 16.6 years (SD = 11.4) in individuals with EAE. Individuals with mild EAE had a mean exposure time of 1444.5 h (SD = 173.3) in 10.9 years (SD = 6.3), those with moderate EAE had a mean exposure time of 2120.2 h (SD = 227.4) in 15.6 years (SD = 8.2) and those with severe EAE had a mean exposure time of 4399.7 h (SD = 416.2) in 25.9 years (SD = 11.9). The increases in severity from none to mild and from mild to moderate were associated with increases in the exposure time of approximately 600 h (Fig. 2).

The exposure time in years and frequency of activity had significant effects on the severity of EAE (Table 3). Each additional year of exposure increased the risk of more severe EAE by a factor of 1.19 or a relative probability of 19%. Each additional day per week increased the risk of more severe EAE by a factor of 4.25, and each additional hour per session increased the risk of more severe EAE by a factor of 3.29. The age at which individuals started surfing was not significantly associated with the severity of EAE.

**Table 3:** Multivariate logistic regression analysis to determine the influence of exposure time, sex and type of sport on the severity of EAE

Predictor	β	*SE* β	Wald	*df*	*p*	*OR*	[95% CI]
Total years	0.18	0.03	36.76	1	0.00	1.19	[0.12; 0.24]
Days per week	1.45	0.33	19.83	1	0.00	4.25	[0.81; 2.09]
Hours per session	1.19	0.37	10.48	1	0.00	3.29	[0.47; 1.91]
Age at start	− 0.01	0.03	0.15	1	0.70	0.99	[−0.06; 0.04]
Sex							
Female	0.29	0.51	0.33	1	0.57	1.34	[−0.71; 1.29]
Male	(Reference)						
Sport							
Kitesurfing	0.86	0.47	3.28	1	0.07	2.36	[−0.07; 1.79]
Kitesurfing + history of windsurfing	0.99	0.52	3.66	1	0.06	2.69	[−0.02; 2.01]
Windsurfing	(Reference)						

*n *= 129, −2 ln *L = *254.530, Chi-square: 98.699, df = 7, *p* < 0.001

*R*^2^: Cox & Snell = 0.535, Nagelkerke = 0.572, McFadden = 0.279

Women had an average severity level of 0.91 (SD = 0.5) and a prevalence of EAE of 59%. Men had an average severity level of 1.67 (SD = 1.1) and a prevalence of EAE of 84%. The mean exposure time was 638.8 h (SD = 301.8) for women and 2509.6 h (SD = 2000.9) for men. Multivariate ordinal regression showed no significant sex differences in the severity of EAE. A trend towards more severe EAE in kitesurfers than in windsurfers was observed, although it was not significant.

A total of 62 participants (48%) reported no otologic symptoms within the past 12 months. Clinically minor symptoms were reported by 44 participants (34%). Symptoms requiring medical treatment were reported by 24 participants (18%), including 14 participants (11%) with additional clinically minor symptoms. Among persons with symptoms, there was a mean of 1.2 (SD = 0.5) minor symptoms per person and a mean of 1.1 (SD = 0.3) symptoms requiring medical treatment per person in the past 12 months. Table 4 shows an overview of the reported symptoms and their frequencies.

**Table 4:** Frequencies of symptoms

	Clinically minor		Clinically significant	
	*n*	%	*n*	%
Water trapping	22	31		
Itching	12	17		
Inflammation	9	13	17	65
Hearing loss	8	11	7	27
Pain from cold water	8	11		
Tinnitus	1	1	2	8
Other	11	15		
Total	71	100	26	100

**Fig. 3:**
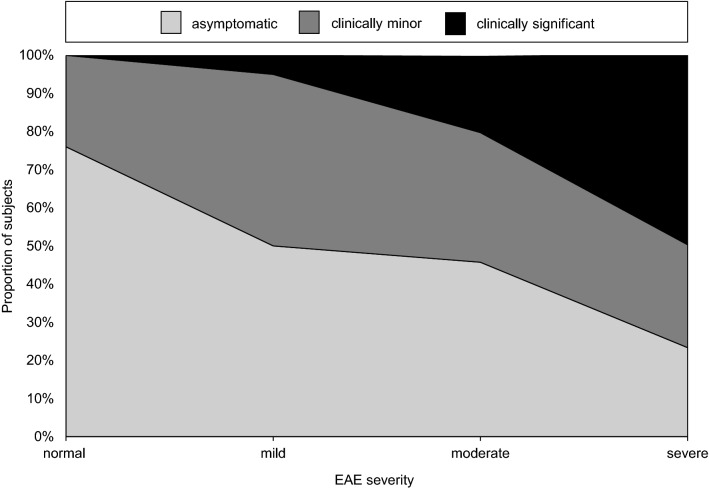
Correlation of the degree of symptoms with the severity of EAE. In cases of multiple symptoms with different severity grades, the more severe symptoms were scored

A significant correlation between the severity of EAE and the degree of complaints was found (*r*_s_ = 0.424, *p* < 0.001, Fig. 3). Participants without EAE reported no complaints in 19 cases (76%). Only 7 participants with severe EAE (23%) were symptom-free. One in five participants (20.0%, *n* = 7) with moderate EAE and one in two participants (50%; *n* = 15) with severe EAE reported symptoms requiring medical treatment in the past 12 months. Individuals with varying degrees of severity of symptoms differed significantly in terms of the severity of EAE (*X*^2^ [2, *n* = 130] = 29.088, *p* < 0.001). Post hoc testing showed that individuals with symptoms requiring medical treatment had significantly more severe EAE than individuals with clinically minor symptoms (*p* < 0.001). Individuals who were asymptomatic and those with clinically minor symptoms did not have different levels of severity of EAE (*p* = 0.385). There was a significant correlation of EAE severity and the number of reported symptoms (*r*_s_ = 0.428, *p* < 0.0001).

## Discussion

The results of the present study indicate a similar prevalence of EAE among wind- and kitesurfers on the German coasts of the North and Baltic Seas and non-professionals engaging in water sports from climatically similar regions (53–89.96%) [6, 9, 10, 37, 40]. In this study, severe EAE with the obstruction of more than two-thirds of the EAC was found in nearly 20% of the participants. Similar prevalences of severe EAE in non-professional surfers were found in the USA (22.2%), Australia (17.4–22.1%) and England (12.4%) [9, 11, 12, 20]. In this study, the age at which the individuals started surfing was not significantly associated with the severity of EAE, which is in accordance with the study by Attlmayr and Smith [9].

The significant positive correlation of exposure time with the severity of EAE shown in previous studies [6–9, 11, 15, 20, 21, 36, 37] was confirmed in this cohort of wind- and kitesurfers. Furthermore, the frequency of activity in days per week and hours per session significantly affected the severity of EAE. Deleyiannis et al. [15] identified significantly more severe EAE in individuals who participated in more than 50 sessions per year than in those who were less active. In other studies, the frequency of activity was not significantly associated with the severity of EAE [21, 37]. Among surfers with at least 10 years of exposure, the prevalences of EAE were 92% in New Zealand [21], 89% in England [6] and 86% in Spain [45], which are very similar to that in wind- and kitesurfers in Germany (92%).

In this study, participants with less than 10 years of exposure had a prevalence of EAE of 61%, which is 1.3 times higher than that reported in a study by Altuna Mariezkurrena et al. [36]. That study reported a prevalence of EAE of 27% in 41 surfers in Spain. Furthermore, the prevalence of EAE of 76.2% in wind- and kitesurfers with a total exposure time of less than 3090 h (*n* = 77) found in this study was 0.7 times higher than the prevalence in surfers in Spain, which was only 46.0% at the same duration of exposure. These differences may be influenced by the higher water temperatures in Spain (12–19 °C). In Ireland, Lennon et al. [10] investigated 119 surfers and found an average of 1909 h of exposure for participants without EAE and an average of 5028 h for subjects with EAE. These values are 1.0 and 1.3 times higher than those for the wind- and kitesurfers in this study. In England, Attlmayr et al. [9] investigated 210 ears of 105 surfers in groups with different total exposure times. They showed a prevalence of moderate to severe EAE in 21.4% of the subjects with less than 2500 h of activity, 23.7% of the subjects with 2500–6000 h of activity and 60% of the subjects with more than 6000 h of activity. When grouped by these exposure durations, moderate to severe EAE was found in 31.2% (*n* = 53), 71.4% (*n* = 40) and 100% (*n* = 15), of the wind- and kitesurfers in this study; moderate to severe EAE was on average 1.1 times more common in wind- and kitesurfers than in surfers.

Nakanishi et al. [35] calculated the exposure time leading to EAE using the product of years and days per week (surfing index) in 373 surfers in Japan. Index values of 70.8 for the obstruction of one-third and two-thirds of the EAC and 82.2 for the obstruction of more than two-thirds of the EAC were reported. These values are 1.8 times higher than the calculated surfing index for wind- and kitesurfers, with values of 20.3 (SD = 10.6) and 36.6 (SD = 17.6) for moderate and severe EAE, respectively. Of note, the water temperatures reported in the study by Nakanishi et al. in Japan were higher than those in the North and Baltic Seas.

Consequently, wind- and kitesurfers have a higher prevalence of EAE and require shorter exposure times to develop the same degree of EAE compared to surfers.

Confirming the gender imbalance in previous studies, the ratio of men to women was 5:1 [6, 7, 9, 10, 36]. As a consequence, the absolute numbers show more cases of EAE in men. However, when considering the exposure time, the risk of developing EAE was comparable between men and women. These results are similar to those of the study by Hurst et al. [8]. As wind- and kitesurfing are increasingly practised by women, it is to be expected that the prevalence of EAE among women may increase.

Although some studies reported differences in the severity of EAE between the right and left ear, in this study, there was no such significant difference. This is in accordance with the findings of numerous other studies [9, 13, 19, 21]. A possible reason for differences in severity based on the side in previous studies was the individual position of the surfer on the surfboard (right or left foot in front) [14, 35]. Other authors considered an influence of evaporative cooling due to a prevailing wind direction as a possible cause [8, 43]. Wind- and kitesurfers usually ride in both directions, so the leg that is in front alternates, resulting in equal exposure of both ears to the wind.

The symptoms reported by wind- and kitesurfers are consistent with those reported by surfers, such as inflammation, water trapping, hearing loss, itching and tinnitus [6–11, 15, 20, 21, 35]. In the previous 12 months, inflammation was the most common symptom. Inflammation was reported by 26 (20%) wind- and kitesurfers and was treated medically in 17 individuals. Eight participants reported that cold water triggered pain. Consequently, this study confirms the finding of Attlmayr and Smith that there is a significant correlation between the degree of complaints and the severity of EAE [9]. The frequency of complaints requiring medical treatment increases significantly with an EAE severity level of 2, indicating the clinical relevance of this stage of ossification.

Wind- and kitesurfing are increasingly popular sports. Kitesurfing will be represented in the Olympics in the future, and windsurfing has been an Olympic sport since 1984. Consequently, awareness of EAE may become increasingly important for ENT specialists and the healthcare system. ENT specialists need to be aware of the high prevalence of this condition in those engaged in water sports. Furthermore, they are encouraged to discuss this issue and possible protective measures with their patients. Even if the effects of protective measures have not yet been conclusively clarified, their use may be recommended [6, 16, 19, 37].

## Limitations

This study is limited by the comparatively small number of participants and may not be generalizable to all wind and kitesurfers. Nevertheless, 130 participants is considered a meaningful sample and represent a cross-section of the investigated group. In participants with a long history of participation, the total exposure time was difficult to assess. However, the interview was very detailed, and episodes involving changing frequencies of activities were taken into account. Furthermore, the reported otologic symptoms were self-reported. A causative relation to the presence of EAE was not proven by clinical examinations. However, all otoscopic pictures were interpreted by an experienced ENT specialist, and no other ear pathologies were reported.

## Conclusion

The prevalence of EAE in wind- and kitesurfers on the German coasts is high at 75.1%. The total exposure time and frequency of activity but not sex influence EAE, which seem to grow more quickly in this population than in surfers. Additional effects of wind and the evaporative cooling of the EAC are believed to explain this phenomenon. The frequency of symptoms requiring treatment increases significantly when more than two-thirds of the EAC is obstructed, suggesting the clinical relevance of this stage of ossification. The results of this study may be used to increase awareness among ENT specialists of the dynamics of EAE and improve their patient counselling.

## Data Availability

Original data are available.
